# Recent Visual Decline—A Health Hazard with Consequences for Social Life: A Study of Home Care Clients in 12 Countries

**DOI:** 10.1155/2010/503817

**Published:** 2010-08-04

**Authors:** Else Vengnes Grue, Harriet Finne-Soveri, Paul Stolee, Jeff Poss, Liv Wergeland Sörbye, Anja Noro, John P. Hirdes, Anette Hylen Ranhoff

**Affiliations:** ^1^Department of Nursing, Diakonhjemmet University College, P.O. Box 184, Vinderen, 0319 Oslo, Norway; ^2^National Institute for Health and Welfare (THL), P.O. Box 30, 00271 Helsinki, Finland; ^3^Department of Health Studies & Gerontology, University of Waterloo, Waterloo, ON, Canada N2L 3G1; ^4^Geriatric Unit, Medical Department, Diakonhjemmet Hospital, P.O. Box 23, Vinderen, 0319 Oslo, Norway; ^5^Institute of Internal Medicine, University of Bergen, 5020 Bergen, Norway

## Abstract

Information about recent visual decline (RVD) and its consequences is limited. The aim was to investigate this in an observational, prospective study. Participants were recipients of community home services, ≥65 years, from Ontario (Canada, *n* = 101618), Finland (the-RAI-database, STAKES, *n* = 1103), and 10 other European countries (the-Aged-in-HOmeCarestudy (AdHOC), *n* = 3793). The instrument RAI-HC version 2.0 was used in all sites. RVD was assessed by the item “Worsening of vision compared to status 90 days ago” and was present in 6–49% in various sites, more common among persons living alone, and in females. In the AdHOC sample, RVD was independently associated with declining social activity and limited outdoors activities due to fear of falling. The combination of stable vision impairment (SVI) and RVD was independently associated with IADL loss. RVD is common and has greater impact than SVI on social life and function. Caregivers should be particularly aware of RVD, its consequences, and help patients to seek assessments, treatment, and rehabilitation.

## 1. Introduction

The prevalence of vision impairment increases in older age groups. In population-based prevalence studies, an estimated 1–14% of those who are 65–74 years old, 7–26% of those who are 75–84 years old, and 30–54% of those who are 85 years and older have visual impairment (VI) [1–5]. The variation may be due to study design, sampling methods, measuring techniques, and the definition of visual impairment and blindness used. 

In Western countries, the most common causes of visual impairment and blindness are age-related macular degeneration (AMD), cataract, glaucoma, and diabetic retinopathy. All of these disorders are more common in advanced age [6, 7]. Refractive error is also a major underlying cause of visual problems, but can be easily corrected with glasses [6]. Reduced peripheral visual fields, depth perception, colour and contrast discrimination, and dark/light adaptation are also common disorders in aging eyes, along with reduced visual capacity [8]. 

Older people are more likely to suffer from chronic diseases, functional and cognitive impairment, and reduced organ reserves [9]. Reduced vision can contribute to these impairments and is associated with depression and a decline in social activity [7, 10], physical imbalance, fear of falling and falls [11–13], and delirium [14, 15]. Visual impairment also lowers the efficacy of patient's rehabilitation after hip fracture [16]. Such problems are known to have complex aetiology and increase the risk of loss of ability to perform instrumental activities of daily living (IADL) and activities of daily living (ADL) [17–20], institutionalisation [11, 21], and mortality [17, 22]. Despite the significant implications on quality of life, visual problems are often ignored by health professionals [23]. 

In this study, we investigated older individuals within the home care setting to determine the prevalence of recent vision decline (experienced within last 90 days) and stable vision impairment (experienced more than last 90 days and still present), and whether recent worsening of vision was associated with changes in social activity, limits in going outdoors due to fear of falling, and IADL loss.

## 2. Methods

### 2.1. Sample and Measurement

The data in this prospective observational study are from three sources: the Ontario RAI-HC database with 101,618 subjects from Ontario, Canada; the RAI-HC database, STAKES, with 1,103 subjects from Finland; and the AdHOC database with 3,793 subjects from European countries: Denmark (*N* = 460), Finland (*N* = 186), Iceland (*N* = 404), Norway (*N* = 384), Sweden (*N* = 244), Germany (*N* = 580), Italy (*N* = 364), France (*N* = 329), The Netherlands (*N* = 193), the Czech Republic (*N* = 365), and the United Kingdom (*N* = 284). The design of the AdHOC study has been described previously [24].

Table 1 provides a summery of information regarding the three databases. All studies collected information with using the Resident Assessment Instrument for home care (RAI-HC) version 2.0 [25]. Subjects under the age of 65 years, those living in institution-like settings, and those with severe dementia were excluded. 

Assessment was done once for each home care recipient. In Ontario, however, the assessments are repeated every six months as part of normal clinical practice. For purposes of this paper, an Ontario prevalence sample was drawn by selecting the first available RAI-HC assessment in July-December 2004 to match the period of data collection for the other two studies. Data were collected by nurses or social workers with specific training for the purpose and with help from the RAI-HC manual [26]. Clients were interviewed and observed, records were explored, and family and home care staff were consulted when necessary. 

The RAI-HC has been validated and tested for reliability and consists of 20 domains with more than 300 clinical items, including sociodemographic data, physical and mental functioning, medical conditions, environmental conditions, and services [27]. The instrument has been translated according to accepted procedures in each participating country.

 Vision was assessed in adequate light with glasses if the patient normally used them. If patient was able to “see fine details, including regular print in newspapers/books”, vision was adequate. Impairments were categorized as 1 (mild; reads large letters but not normal type in newspapers and books), 2 (moderate; cannot read newspaper headlines, but recognizes objects), and 3 (severe/very severe probably unable to recognize objects, but eyes can follow moving objects/no vision or can only see light, colours, or contours) (score 1–3). The RAI-HC provides question about vision decline; “Worsening of vision compared to status 90 days ago”. If client answered “Yes”, then recent vision decline (RVD) was present. Stable vision impairment (SVI) was present, when patient scored 1–3 on vision and “No**”** on “Worsening of vision compared to status 90 days ago”.

The RAI-HC contains variables related to social life, for example, enjoying spending time with others, in conflict with others, time spent alone, feeling lonely, and changes in social activity. Decline in social activity is defined as decline in the level of participation in social, religious, occupational, or other preferred activities within the past 90 days. The level of participation refers to the number of different types of social activities, the frequency, and the quality of relationships [26]. 

 The RAI-HC includes several subscales. The Cognitive Performance Scale (CPS) is scored 0–6 (0 = normal, 6 = severe cognitive impairment) [28]. The Depression Rating Scale (DRS) is scored 0–14 and depression is probably present if the score is 3 or more. The DRS consists of seven items; sadness, persistent anger, unrealistic fears, repetitive health complaints, other repetitive concerns, worried facial expressions, and crying during last three days [29]. Activities of Daily Living (ADL) considered were personal hygiene, toilet use, locomotion, and eating. Each item was scored from 0 (independent/set-up help only) to 4 (total dependence), and they were combined in the form of the ADL Hierarchy Scale [30] with a range 0–6. Loss of ADL was considered to be present for persons with a score of greater than 1 on the ADL Hierarchy Scale. Instrumental Activities of Daily Living (IADL) were assessed according to meal preparation, ordinary housework, managing finances, managing medications, managing telephone use, shopping, and ability to get to places beyond a walking distance. Each item was scored 0 (independent) to 3 (dependent on others) and was then summed to create the IADL Involvement Scale with scores from 0 to 21 [30]. IADL loss was considered when the IADL Involvement Scale had scores greater than or equal to five. 

Fall history, emergency calls and hospital admissions refer to events in the past 90 days. Polypharmacy was defined as taking five or more different medicines [31]. The assessors recorded the disease diagnoses from nursing documents, by patient self-report and proxy information. Informal caregivers were relatives, neighbors, or friends. “Any personal services” were visiting nurse and home care providers who supported the client in ADLs, special treatments and therapies, and performed nursing procedures. “Home making help” was home help which primarily assisted the client in IADLs. 

Informed consent from all participants and ethical approval according to each country's regulations and laws were obtained in the AdHOC study. It is census level information based on research permission by the Ministry of Health and Welfare in Finland. In Ontario, the RAI-HC is completed as part of normal clinical practice. Deidentified RAI-HC data compliant with national privacy standards are available to interRAI researchers according to an existing agreement with the Canadian Institute for Health Information.

### 2.2. Statistical Analyses

The statistical analyses from the Ontario database were done in Canada and from the STAKES database in Finland using SAS version 9.1 (SAS Institute, Cary, North Carolina), while the AdHOC data were analysed in Norway using the Statistical Package for Social Sciences (SPSS 15.0, 2008) [32] and prepared as described by Peat and Barton [33]. 

Frequencies were calculated for the variables describing client self-reported categories of visual function and visual impairment, stable and changed, through last 90 days for each country contributing data to this study. 

For the AdHOC-data only descriptive statistics were computed for the variables describing client characteristics. Analyses to find differences in characteristics between subjects with adequate vision (AV) and subjects with SVI, differences between subjects with AV and subjects with RVD, and differences between subjects with SVI and subjects with RVD were done. 

Pearson's Chi-square test for categorical variables and *t*-tests for continuous variables were used. Odds ratios are presented with 95% confidence intervals for categorical variables and Standard error difference (SE diff.) for continuous variables; variations between SVI and RVD. Change in social activities, limits on going outdoors due to fear of falling, and IADL loss were selected as dependent variables in three multivariate logistic regression models. Independent variables (explanatory) were dichotomized SVI/RVD and presence/absence of the characteristics of age ≥82 years (mean age), female gender, living alone, visual impairment (VI), no cognitive impairment, feeling lonely, unsteady gait, feeling dizzy, falls, limits on going outdoors due to fear of falling, ADL loss, IADL loss, any informal care, any personal services, polypharmacy, and hospital episodes. The chi-square tests were run to reveal the association to each of the dependent variables. Bivariate logistic regression analyses were used to identify variables for inclusion in the multivariate regression models. If the independent variables were significant (*P* < .05) in any of the bivariate logistic regression analyses, they were tested in each of the logistic regression models (change in social activity, limits on going outdoors due to fear of falling, and IADL loss). The explanatory variables were entered into the logistic regression models with a sequential method, one at a time, using results from a literature review, and by the strength of the correlation with the outcome variable. 

The statistics are presented as percentages, chi-square (*P*-value) and odds ratios with 95% confidence intervals, regression coefficient (*b*), and Nagelkerke *R* square (*R* ^2^) in the text. Odds ratios were executed to estimate the probability for a categorical response variable with two outcomes. Nagelkerke *R* square test was performed to indicate the percentage of variation in the actual dependent variable, explained by the independent variables. A goodness-of-fit test of the null hypothesis, the Hosmer and Lemeshow test, was used to show how adequately the model fits the data. 

## 3. Results

### 3.1. Prevalence of Visual Impairment and Recent Visual Decline

**Figure 1:**
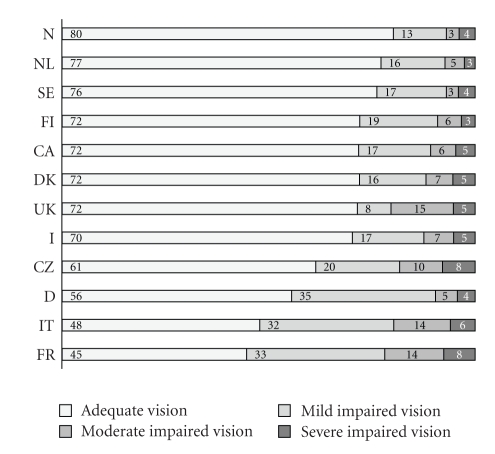
Vision (based on self- and proxy reports and assessed by a simple reading test) in older people receiving homecare in 12 different country sites (%); *N* = 106328.

**Figure 2:**
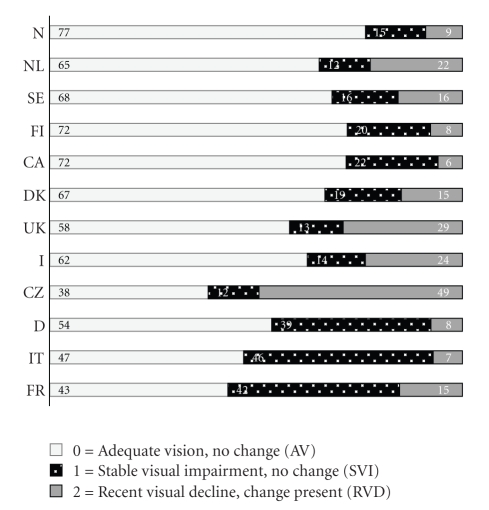
Self-reported visual impairment, stable and changed, through last 90 days in older people receiving homecare in 12 different country sites (%). *N* = 106328.

The prevalence of VI ranged from 19.8% (Norway) to 55.3% (France) (Figure 1). RVD was found in 5.9% (Canada) to 49.3% in the Czech Republic (Figure 2). In the AdHOC study cataract and glaucoma were more frequent when clients with RVD were compared with those with SVI, (OR = 3.4, 95% CI 2.7, 4.3, *P* < .0001; and OR = 2.2, 95% CI 1.6, 3.1, *P* < .0001, resp.). Of all subjects with cataract and glaucoma, 45.8% and 49.7%, respectively, were able to read normal print. As for the RVD subjects, 36.0% with cataract and 27.0% with glaucoma were able to do so. 

**Figure 3:**
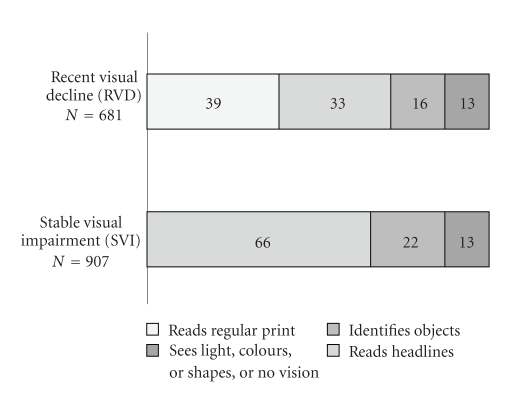
Visual ability in two groups of older homecare clients: stable visual impairment and recent visual decline (for the period of the last 90 days) (%); *N* = 3793.

Figures 1 and 2 are showing categories of VI and change in vision for each country contributing data to this study. Figure 3 shows categories of VI in subjects with RVD and SVI from the AdHOC study.

### 3.2. Association between Recent Visual Decline and Social Life

Table 2 presents differences among clients in the AdHOC study. Subjects with RVD more often withdrew from activities, had reduced social interactions and felt lonely. They were also more alone and complained of more mood disturbances compared with clients with SVI. Depression symptoms were also more common in the RVD group compared with those with SVI, expressed by items in the DRS scale: feelings of sadness (OR = 1.8, 95% CI 1.5, 2.3, *P* < .0001), pained or worried facial expressions (OR = 1.4, 95% CI 1.1, 1.7, *P* = .013), and recurrent crying or tearfulness (OR = 1.5, 95% CI 1.1, 2.0, *P* = .04). Change in social activity occurred among 51.0% of the RVD participants during the past 90 days and 44.4% felt distress from this. There were differences between the country sites as data from the Netherlands, Norway, and Sweden showed no differences between the subjects with RVD and SVI on these social parameters. 

A logistic regression model for decline in social activity included the variables older age (≥82 years) (mean age), female gender, RVD, DRS ≥3 (depression most likely present), polypharmacy, and falls. RVD was the strongest independent variable in the model associated with social change (OR 2.3 95% CI 1.8–2.8, *P* < .0001; Nagelkerke *R* ^2^ = 0.108; Hosmer and Lemeshow test Chi-square 7.347, df 8, significance of the test: 0.500).

### 3.3. Associations between Recent Visual Decline and Limits on Going Outdoors due to Fear of Falling

Limits on going outdoors due to fear of falling were reported by the majority of the RVD clients (60.5%). Unsteady gait, feeling dizzy, polypharmacy, falls, experiencing poor health, and living alone were more common among the RVD clients compared with those who had SVI (Table 2). Subjects with RVD who reported limits on going outdoors due to fear of falling had experienced falls more often (OR 1.7; 95% CI 1.3, 2.1, *P* < .0001) compared with those with SVI. When analysing each country separately, there were little variations between SVI and RVD for the mentioned variables, but when analyzing data from all the 11 European countries together the power increased and the differences became significant. 

A logistic regression model for limits on going outdoors due to fear of falling included older age (≥82 years), female gender, RVD, unsteady gait, and DRS ≥3 (depression most likely present). Unsteady gait (OR = 16.7 Cl 12.6–22.1, *P* < .0001) showed the strongest association, followed by RVD (OR = 1.3; 95% CI 1.0–1.6, *P* = .042, Nagelkerke *R* ^2^ = 0.394; Hosmer and Lemeshow test; Chi-square 6.459, df 8 and significance of the test: 0.596).

### 3.4. Associations between Recent Visual Decline and Instrumental Activities of Daily Living

The RVD clients were more independent in all the IADL items compared with clients with SVI, except for transportation. However, those with RVD received more home making help (Table 2) and “meals on wheels” (OR 1.7, 95% CI 1.4–2.3, *P* < .0001) compared with those with SVI. 

When comparing subjects with RVD with those with adequate vision (AV), the RVD subjects were more dependent in all IADL items, except telephone use. When comparing all subjects with VI (RVD and SVI) to those with AV, the VI subjects were more dependent in all IADL items. 

A logistic regression model for IADL loss included older age (≥82 years), female gender, VI, cognitive impairment, unstable gait, and depression. The strongest variable in the model was VI and cognitive impairment (OR = 2.3, CI 2.0–2.7, *P* < .0001; and OR = 4.1, CI 3.5–4.7, *P* < .0001, resp.; Nagelkerke *R* ^2^ = 0.241; Hosmer and Lemeshow test; Chi-square 7.561, df 8, significance of the test: 0.478).

## 4. Discussion

Recent visual decline was common among older home care clients in study populations from Canada and 11 European countries. Female gender, and living alone were associated with RVD compared to clients without visual impairment or with stable visual impairment. Cataract and glaucoma were more common when RVD was present (Table 2). This study also demonstrates that RVD has a significant impact on the social life and function of older people. 

Because vision impairment is common, increases by age, and affects a significant number of people who receive health care services, these findings are important. Since there are no previous studies of recent visual decline in older people, our findings cannot be compared with others. However, studies of visual impairment in older people without discriminating between SVI and RVD have also demonstrated an association with older age, eye diseases, functional and social problems, depression, and increased risk of fear of falling and falling [11–13, 17–19].

The most important findings in our study are that older persons with RVD have even more functional problems are less socially active than those with SVI, and have an increased risk of isolation, depression symptoms, and dependency. These persons are vulnerable and need special attention.

RVD was independently associated with change in social activities. The reason for this might be the reduced ability to orientation in the environment. Problems being able to recognize familiar faces may be embarrassing and cause isolation. Also, fear of falling can be caused by RVD and contribute to reduced social and physical activity, subsequently leading to reduced muscle strength, balance, and endurance, thereby increasing fall risk. This is supported by our results, showing that RVD clients had more falls during the past 90 days than SVI clients. RVD was independently associated with limits on going outdoors due to fear of falling in logistic regression analysis, contributing to reduced activity. 

Indicators of poor health (polypharmacy, depression symptoms, emergency calls to the home, recent hospital day episodes, and self-reported poor health) were more common in the RVD subjects than in those with SVI. The RVD subjects received more informal help and home making help, but not more formal personal services (visiting nurse and home caregivers) than the SVD, although their health was poorer. Clients may have been able to perform ADLs by learned routine, but were in need of assistance to perform IADLs where vision was essential. Previous studies demonstrated that IADL problems in doing ordinary housework, preparing hot meals, shopping, and getting to places beyond a walking distance are associated with visual impairment [17–20]. In this study, such problems were less common among those with RVD compared with clients with SVI, but more common than in those with AV. Visual impairment (RVD and SVI) was independently associated with IADL loss in a regression analysis. This may indicate that clients with RVD are able to perform some IADL by routine, but become more dependent over time without proper treatment and rehabilitation or an increase in need of help has not be registered. 

The method used in this study depended on patient- or proxy-reported recent visual decline. Assessments of vision were performed by a simple reading test and the validity of the assessment of visual impairment is considered to been reasonably good. Information about whether this impairment had started or worsened over the previous 90 days is more uncertain. The use of proxy information to improve the validity of information about the health and functional status of older persons is somewhat questionable, but widely used in clinical practice [34]. There might also be cultural differences in what people report in self-reported health interviews that could influence the results of this multinational study [35].

Another possible limitation is the larger number of assessors involved. Even with compulsory training, there might be individual differences in performing the assessment. However, assessment of visual impairment and the other items of the RAI-HC have a fairly good interrater reliability [25].

The study was not particularly designed to investigate RVD, and data are retrospective regarding vision impairment. Only a simple reading test was used to detect visual impairment. A battery of tests is needed to detect visual impairments in older people, including self report [8]. Laitinen et al. demonstrated a correlation between self-reported visual ability and measured visual function [5]. Self-reported visual disturbances include not only visual disorders resulting from refractive errors, but also disturbances regarding contrast sensitivity, glare sensitivity, stereopsis, and visual fields [36]. This is supported by our study in that several of those with RVD were able to read regular print in the newspaper, but reported poorer vision over the past 90 days. Some of the reported difficulties may not be entirely visual, but could be caused by, that is, cognitive problems, a phenomenon believed to be present in this age group [37]. Prospective vision tests combined with self-reports, with intervals of three months, for example, may be the best methods to detect RVD and are recommended for further studies.

The differences in the prevalence of RVD and VI between countries in this study correspond to differences in other functional parameters illustrating that there are major differences in the characteristics of home care users between geographical regions [24]. Laitinen et al. showed that cognitive capacity was one of the most important factors affecting the use of eye care services [38]. In our study, the sites with the highest prevalence of VI and RVD (see Figure 2) had the highest mean score on CPS [24], indicating cognitive impairment. In Ontario, a comprehensive evaluation with the RAI-HC instrument is completed regularly as part of normal clinical practice. VI is most likely discovered and treated and may partly explain the smaller percentage of clients with RVD compared to the other sites. Also, information collected in one home care site is believed to be representative for home care clients in that particular site, and not for the entire country. Another aspect is that data represent home care clients from different country sites with unlike selection criteria for these services. Data from the participating sites differed in physical and mental functioning values and also in providing formal and informal healthcare. These aspects are presented in other publications and reflect the different healthcare systems and welfare models [24, 39]. 

One important implication of our findings is that recent changes in social activity, limits on going outdoors, and difficulties in performing IADL may be indicators of visual decline, and should trigger an assessment by a nurse or a physician. Most likely, many seniors with RVD are unfamiliar with the actions necessary to improve visual function, avoid obstacles, or move around safely. Informal care-givers should be informed about RVD and its consequences for social and functional life. Decreased social activity must be considered a sign of isolation and a risk that their home is turning into a prison [39]. In this respect, interventions to improve vision and prevent the unwanted consequences of recent visual decline are believed to be important, but must be studied.

Regular assessments of visual function and the eyes are recommended for older persons. Many have treatable eye disorders and may benefit from education, coaching, treatment, and remedies to improve vision [7, 10, 23, 38].

## 5. Conclusions

The prevalence of recent visual decline (within 90 days) in older home care clients was high, between 5.9 and 49.3% in various sites. Recent visual decline may have more severe consequences than stable vision impairment, particularly in terms of changes in social activity and fear of falling. RVD is associated with dependency in IADL, but less than with stable visual impairment. Nurses, as in this study, should be particularly aware of RVD and its consequences and help the patients to receive proper assessments, treatment, and rehabilitation. Also, informal care-givers should be provided with this information.

Whether such an intervention will reduce the impact on social activity, fear of falling, and loss of IADL is unknown and should be investigated in future studies.

## Figures and Tables

**Table 1:** Comparison of the three databases.

	Database		
	Ontario RAI-HC data base, University of Waterloo, Canada	RAI-database STAKES, Finland	AdHOC study, Universita Cattolica de Sacro Cuore, Italy

Countries included	Canada	Finland	Czech Republic, Denmark, Finland, France, Germany, Iceland, Italy, The Netherlands, Norway, Sweden, United Kingdom

Specific locations within countries	Ontario	Five home care agencies serving urban area in different parts of Finland	One or several home care agencies serving a urban area, in each of the countries

Criteria for selecting the homecare agencies	All in the province	Volunteer agencies	Volunteer agencies

Inclusion criteria for the homecare clients	Every client assessed once over a given time frame	Every client assessed once over a given time frame	Random sample assessed once over a given time frame

Refusal rates	None	None	0–57%

Purpose for collecting data	Care planning, quality monitoring, and administrative decision making for homecare	On-going national initiative to improve statistics and quality of care within the elderly care services	Research study with a coaim to construct a European data base for home care

Collection of data	January 2004–December 2004	July 2004–December 2004	August 2001–December 2002

Number extracted for the current analysis	101.618	1.103	3.793

Education of the data collectors	Standardised programs, ≥8 hours	Standardised national programs, ≥8 hours	Standardised programs, ≥8 hours

Funding	Ontario Ministry of Health and Long-Term Care	Local authorities and STAKES	European Union (5th framework)

**Table 2:** Characteristics of patients in the AdHOC study in relation to self-reported vision; adequate vision (AV), stable visual impairment (*S* *V* *I*)^*a*^, and recent visual decline (*R* *V* *D*)^*b*^ (percentage of cases unless otherwise stated). *P* values indicate differences between the groups.

	All	AV	SVI	RVD	*P*	*P*	*P*	OR/SE diff.
					AV vs. SV	AV vs. RVD	SVI vs. RVD	(95% CI) SVI vs. RVD
Overall number (%)	3793(100)	2205(58.1)	907(23.9)	681(18.0)				
Demographic variables age (year)								
Mean (SD) min.-max. 65.1–104.5	82.3 (7.3)	81.5 (7.3)	83.5 (7.5)	83.2 (7.2)	<.0001	<.0001	.390	0.37 (0.4–0.7)
<75	17.4	66.5	19.4	14.1	<.0001	<.0001	.795	1.0 (0.7–1.3)
75–84	43.8	60.3	22.2	17.5	.014	.221	.388	1.1 (0.9–1.3)
≥85	38.9	52.0	27.9	20.1	<.0001	<.0001	.500	0.9 (0.8–1.1)
Female gender	74.2	57.5	23.4	19.1	.699	.04	.005	1.4 (1.1–1.8)
Lives alone	61.6	60.2	20.5	19.3	<.0001	.259	<.0001	1.8 (1.4–2.2)
Physical functioning								
Hearing impairment	37.1	43.9	32.3	23.8	<.0001	<.0001	.701	1.0 (0.8–1.2)
ADL^c^ loss Hierarchy Scale (0–6)								
Mean (SD) min.-max. (0–4)	0.6 (1.2)	0.5 (1.0)	1.1 (1.4)	0.4 (1.0)	<.0001	.088	<.0001	0.06 (0.6–0.8)
ADL Hierarchy Scale (score >0)	27.2	46.8	39.7	13.6	<.0001	<.0001	<.0001	0.5 (0.4–0.6)
IADL^d^ loss Involvement Scale (0–21)								
Mean (SD) min.-max. (0–21)	5.1 (2.9)	4.5 (2.7)	6.3 (3.0)	5.5 (3.0)	<.0001	<.0001	<.0001	0.15 (0.5–1.1)
IADL Involvement Scale (score ≥5)	58.3	49.8	30.5	19.6	<.0001	<.0001	<.0001	0.6 (0.5–0.7)
Mental functioning								
Cognitive Performance Scale CPS (0–6)								
Mean (SD) min.-max. (0–5)	1.0 (1.4)	0.8 (1.3)	1.6 (1.7)	1.0 (1.4)	<.0001	<.0001	<.0001	0.08 (0.4–0.7)
Cognitive impairment CPS scale ≥1	45.8	49.4	31.0	19.6	<.0001	<.0001	<.0001	0.7 (0.6–0.8)
Change in cognition within 90 dd	6.4	43.4	38.1	18.4	<.0001	<.0001	.011	0.6 (0.4–0.9)
Depression Scale, DRS Scale (0–14)								
Mean (SD) min.-max. (0–14)	1.1 (2.0)	0.9 (1.8)	1.3 (2.1)	1.5 (2.4)	<.0001	<.0001	.014	0.11 (0.5–0.1)
Depression symptoms (DRS scale ≥3)	16.1	46.5	27.3	26.2	<.0001	<.0001	.013	1.4 (1.1–1.7)
Mood decline within 90 dd	11.5	47.5	25.3	27.2	.019	<.0001	.003	1.5 (1.1–2.0)
Social functioning								
Withdrawal from activities	14.4	46.7	24.6	28.6	.012	<.0001	<.0001	1.7 (1.3–2.2)
Reduced social interactions	20.6	50.7	25.7	23.6	.007	<.0001	.021	1.3 (1.0–1.6)
Social activity, changed	34.7	53.0	20.6	26.3	.349	<.0001	<.0001	2.4 (2.0–3.0)
Feelings of loneliness	21.3	52.0	20.2	27.8	.484	<.0001	<.0001	2.3 (1.8–2.8)
Conditions								
Dizziness	23.5	53.6	18.6	27.8	.035	<.0001	<.0001	2.6 (2.0–3.2)
Unsteady gait	62.2	54.1	25.3	20.6	<.0001	<.0001	.025	1.3 (1.0–1.6)
Limits on going outdoors	47.6	50.8	26.4	22.8	<.0001	<.0001	.002	1.4 (1.1–1.7)
Falls Mean (SD) min.-max. (0–12)	0.6 (1.4)	0.5 (1.3)	0.7 (1.6)	0.7 (1.5)	.010	<.0001	.410	0.08 (−0.2–0.1)
Any falls within 90 dd	25.7	50.5	26.3	23.3	<.0001	<.0001	.029	1.3 (1.0–1.6)
Experienced health								
Poor experienced health	30.5	51.0	23.8	25.2	.044	<.0001	<.0001	1.7 (1.4–2.1)
Medications								
Mean (SD) min.-max. (0–20)	5.4 (2.9)	5.3 (2.9)	5.3 (2.9)	6.1 (2.6)	.614	<.0001	<.0001	0.14 (1.2–0.6)
Polypharmacy (≥5 daily)	62.4	55.9	23.3	20.8	.715	<.0001	<.0001	1.7 (1.4–2.1)
Use of services								
Informal caregiver	70.3	54.7	26.2	19.2	<.0001	<.0001	.374	0.9 (0.7–1.1)
Any personal services	68.9	56.3	25.4	18.3	<.0001	.077	.190	0.9 (0.7–1.1)
Homemaking help	47.3	60.5	20.3	19.2	<.0001	.565	<.0001	1.5 (1.2–1.9)
Occupational therapy	2.4	57.1	23.1	19.8	.943	.673	.676	1.1 (0.6–2.2)
Emergency services at home	5.5	49.5	23.6	26.9	.390	<.0001	.025	1.6 (1.1–2.3)
Hospital episodes, over nights	13.6	50.7	29.7	19.6	<.0001	.039	.273	0.9 (0.7–1.1)
Hospital episodes, days	5.0	51.6	20.0	28.4	.752	<.0001	.002	2.0 (1.3–3.0)
Increase in care needs within 90 dd	6.3	55.9	25.2	18.9	.540	.585	1.0	1.0 (0.7–1.5)
Cataract	20.0	45.8	19.0	35.2	.923	<.0001	<.0001	3.4 (2.7–4.3)
Glaucoma	7.7	49.7	19.9	30.5	.852	<.0001	<.0001	2.2 (1.6–3.1)

^a^SVI = impaired vision not changed through the last 90 days, ^b^RVD = vision declined through the last 90 days, ^c^ADL = activities of daily living, and ^d^IADL = Instrumental activities of daily living.
